# A Comparison of Working Conditions and Workers’ Perceptions among On-Site, Telework, and Hybrid Workers in Ecuador during the COVID-19 Pandemic

**DOI:** 10.3390/ijerph192114337

**Published:** 2022-11-02

**Authors:** Andrea Vinueza-Cabezas, Gabriel Osejo-Taco, Alejandro Unda-López, Clara Paz, Paula Hidalgo-Andrade

**Affiliations:** 1 Escuela de Psicología y Educación, Universidad de Las Américas, Quito 170124, Ecuador; 2 Universidad Latina de Costa Rica, San José 11501, Costa Rica

**Keywords:** telework, hybrid work, on-site work, COVID-19, perceive productivity, workers’ perceptions, working conditions

## Abstract

The COVID-19 pandemic has forced many companies to adopt different work modalities to ensure their operation during this period. In this study, we described and compared working conditions and perceptions among face-to-face workers, teleworkers, and hybrid workers in Ecuador. A cross-sectional study was conducted with a sample of 542 participants, using a self-report survey to assess sociodemographic data, working conditions, and workers’ perceptions. Variables were described and then compared by the Chi-square test, ANOVA, and the Kruskal–Wallis test. The results indicated a higher proportion of on-site workers without higher education and in the public sector compared to the other modalities. At the same time, there was evidence of increased perceived productivity. People in the hybrid modality tended to have more than one job, earning a higher monthly salary, perceiving a decrease in productivity, an increase in daily working hours, and a lower capacity for time management. In addition, most teleworkers reported fair working conditions, a dedicated workspace, and easy adaptation to this work mode. This study builds a more in-depth understanding of how workers perceived their working conditions among work modalities for organizational decision-making because the evolution of the COVID-19 pandemic is modifying the ways of working permanently.

## 1. Introduction

In January 2020, the world experienced a drastic change in how we lived and worked due to the rapid spread of the SARS-CoV-2 virus, which caused a worldwide public health crisis [1]. As it was an unknown virus, and due to the lack of treatments and vaccines, most countries implemented isolation measures to avoid contagion and saturation of the health system [2,3]. In Ecuador, the authorities declared a state of emergency on 16 March 2020 [4]. The measures established a lockdown and the suspension of on-site work for all public and private sector workers and employees, except those providing public services, health, security, financial, provision of food, medicines, medical and sanitary supplies, transportation, and strategic sectors that helped to combat the spread of COVID-19 [5]. On 15 May, the Ecuadorian government created a color-coded alert system to inform the public about the epidemiological situation of each province. This system also included specific guidelines and information on activities that could and could not occur in person [6]. On 22 June, the Ley Orgánica de Apoyo Humanitario (Organic Law of Humanitarian Support) was issued to establish the necessary support measures to face the aftermath of the health crisis caused by COVID-19. The following labor measures were established to preserve jobs and guarantee work stability: (1) the reduction of the working day with salary proportional to the number of hours worked; (2) the creation of a type of emergency labor contract with a maximum term of two years for the urgent hiring of personnel to increase production or to comply with pending obligations; (3) employers were allowed to unilaterally organize the employees’ vacation schedule or compensate for absence from work as vacation time already taken; (4) access to unemployment insurance for workers affiliated to the Ecuadorian Social Security Institute; (5) priority hiring of workers, professionals, goods, and services of local origin; and (6) job stability for health workers who have worked during the coronavirus health emergency (COVID-19) with an occasional contract or provisional appointment in any position in any health care center of the Integral Public Health Network [7]. From May to June 2020, the unemployment rate reached 13.3% at the national level [8], decreasing to 5% at the end of the year [9]. In 2021, the national unemployment rate was 5.2% [10], and in January 2022, it was 5.4% [11]. The informal employment rate was 51.1% in 2020 [9], and it fell to 49.5% in 2021 [10]. As of February 2022, the informal employment rate increased to 53.1% [11], which shows that self-employed people account for a higher percentage of the working population in Ecuador.

This situation caused health and economic problems for organizations and their employees, drastically altering their daily routines and work practices [3]. Many companies faced the need to sustain the economy and ensure the sustainability of their business and work activities during this period by maintaining on-site work when necessary and by implementing new work modalities such as telework and hybrid work [1]. 

Several recent studies examined the experience of working remotely in the context of the COVID-19 pandemic. However, there is a lack of studies comparing work modalities. In the present study, we aimed to fill this gap by describing and comparing employees’ sociodemographic characteristics, working conditions, and perceptions of their performance between on-site, telework, and hybrid work during the COVID-19 pandemic in Ecuador.

During the COVID-19 pandemic, all necessary activities for the continuity of life and society were categorized as essential, which caused on-site jobs to remain active. On-site work refers to workers always occupying the same physical space, for example, in a company building [12]. Many professionals who worked on-site during the pandemic were blue-collar and essential or frontline workers [13,14]. Frontline occupations included healthcare practitioners; technical healthcare support; protective services; food preparation and serving; building and grounds cleaning, maintenance; personal care and service; farming, fishing, and forestry; construction and extraction; installation, maintenance, and repair workers; production and transportation; and material moving [15,16,17].

Wang et al. [18] identified several factors that attracted employees to prefer on-site work, such as better workplace setup, in-person meetings, interactions with colleagues, and working on specific tasks in the office. However, during the pandemic, these workers faced much higher risks than those traditionally encountered in their occupations. Essential workers who worked on-site risked infecting themselves and their loved ones; however, staying at home meant not being able to work or support their household [14]. In order to protect their workers, prevent the spread of the virus, and assure the continuity of their operations, companies included preventive measures. Some of these were: mask use, hand hygiene, ventilation, physical distancing at work, COVID-19 testing for workers [19], use of hand sanitizer, increasing the frequency of surface cleaning, periodic checking of workers for signs of COVID-19, and application of epidemiological fencing with colleagues and family members [20]. Several studies examined the engagement and productivity of on-site workers during the COVID-19 pandemic. For example, one study from Poland found that on-site workers reported no significant differences in their work engagement compared to the other work modalities [21]. Another study from Argentina shows low work satisfaction among hospitalist doctors [22]. In terms of productivity, a study conducted in China found that on-site work is more productive than remote work [23]. Another study from the United Kingdom reported that on-site construction workers claimed that their productivity improved even though some construction projects reduced overall productivity and pushed back the schedule [8]. However, more studies are still needed, given that results vary and are still inconclusive [21,24].

On the other hand, some jobs migrated to telework. Telework is a modality of work in which the employee works outside the employer’s workplace, in various locations such as satellite offices or at home, using information and communication technologies (ICTs) [1,25,26,27,28,29,30]. Telework was first introduced during the oil crisis in the 1970s [31]. Since then, this flexible work structure has begun to gain popularity as an alternative to the traditional work style [2]. Telework is more common in knowledge-intensive services, given that several high-skill jobs use ICTs such as laptops and cell phones [15]. On the other hand, it is less common in the manufacturing and construction sectors and low- and medium-skilled occupations [15].

During the COVID-19 pandemic, many employees were suddenly and unexpectedly forced to telework. This change mostly came without the proper preparation, planning, training, or the necessary equipment and tools to work from home [1]. In turn, they had to designate or improvise a space to work within their homes: dining tables became desks, sofas became office chairs, and living rooms and dining rooms became communal workspaces [32]. In turn, teleworkers faced challenges such as balancing work with childcare, family relationships, domestic responsibilities [32], a lack of clarity about tasks and priorities, uncertainty about where to obtain specific support, and handling different or new technological tools [15]. Moreover, organizations needed to quickly transition to ICTs to replace face-to-face services, using platforms such as Zoom or Microsoft Teams, and to implement conditions that allow individuals to work remotely without adequate training or support, regardless of their previous teleworking experience [2,33].

Despite the abrupt transformation, recent studies from different countries found positive consequences of this modality change, such as having flexible working hours and removing geographical barriers by working from other locations [34]. For example, in Portugal, although most teleworkers experienced this modality for the first time, they adapted easily and quickly to it [35]. Another study in Kuwait showed that most teleworkers considered they were more productive working from home, appreciated the flexibility in setting work schedules, and reported satisfaction with the company’s immediate response to COVID-19 remote working conditions [36]. Similarly, a study from South Africa revealed that teleworkers, provided with the right technological resources and teleconferencing training, enjoyed a better work–life balance, reported more productivity, and saved money [37]. In contrast, some studies show negative implications. For example, Tavares and colleagues [35] reported that teleworkers often work very late to compensate for productivity losses due to household chores, childcare, and time spent on social networks. It was also found in the study conducted in Ecuador that those who telework more than 8 h a day experience higher levels of work–family conflict [38]. On the other hand, in a study in Japan, teleworkers reported an increase in work hours and meeting hours [39]. A similar study conducted during the confinement in Chile revealed that for most academics, telework meant working more hours than the official time commitment needed to achieve high-quality results and that online classes and meetings demanded more preparation and work [40]. Moreover, research by Sandoval-Reyes and colleagues, whose respondents are mostly from Colombia and Ecuador, found that the demands of telework increased perceived stress and reduced work–life balance and work satisfaction [41]. Other studies examined the effects of telework on productivity and found inconsistent results. For instance, research conducted in the US revealed an increase in self-reported work productivity over time for those who shifted to telework since the outbreak of COVID-19. Furthermore, income and lifestyle had a moderating effect on the ability to adapt to telework. A probable explanation is that a higher income translates into greater access to resources and the ability to make appropriate adjustments, such as upgrading Internet infrastructure [42]. Another study conducted in Jordan found that most engineers who teleworked during the pandemic did not notice any change in their productivity [24]. Similarly, a study conducted in Ecuador on a sample of 459 teleworkers in the public educational sector showed that most teleworkers reported stable levels of productivity. The authors remark that this comes as a consequence of developing competencies that have a positive impact on productivity, such as self-motivation, self-discipline, and organization [43].

Vaccine coverage and knowledge about COVID-19 prevention and treatment have advanced. It has allowed countries’ authorities to lift isolation measures and implement a hybrid work modality. This method allows employees to plan their workday and carefully select the most appropriate workspace (office or home) based on their activities [12,44]. Companies face the challenge of adopting a hybrid work model and maintaining productivity through “restructuring the working process, identifying tasks, redesigning workspaces, and relocating offices” [14]. In turn, hybrid work offers savings in-office maintenance costs, greater flexibility in hiring talent from different geographic areas, and even staff with disabilities [45]. For employees, it provides higher flexibility in adjusting their working hours according to their personal and family needs [14]. Moreover, it allows them to work where and when they are most productive, demonstrating their strengths and increasing their productivity [45]. However, adopting hybrid work practices depends on the nature of the tasks; generally, white-collar jobs can do so, while those involved in manual and physical labor are not usually suitable for this modality [14].

The research on the effects of hybrid work on employee conditions is currently in its early stages. However, it shows results for productivity, satisfaction, and implementation recommendations. For example, a study conducted in Hungary [46] found that full-time telework is not recommended for organizations that rely on Generation Z talent. The same research suggests that hybrid work should consider a weekly time allocation for on-site work and informal business meetings and build new communication and behavioral practices for the leader to avoid conflicts due to misinterpretation of non-verbal cues in virtual environments. In another study in Kuwait, the authors found that on a hybrid modality, most employees reported they could effectively meet their work expectations as conditions at home are more favorable and more satisfactory than at the office [36]. Furthermore, a study conducted in China in the spring of 2020, a time when companies started to reopen their offices and their employees transitioned to a hybrid work mode, found that the participants perceived higher productivity and higher satisfaction in hybrid work compared to telework full-time [18]. The COVID-19 pandemic has accelerated the transition to the next phase: a hybrid model of work and workspace, expected to continue functioning as a work option for businesses and employees when the pandemic ends in the future [47]. 

Although many recent studies examined the remote work experience in the context of the COVID- 19 pandemic, there is a lack of studies that focus on comparing the characteristics of the people working in each of the presented modalities: on-site, telework, and hybrid work. In order to address this gap, the current study aimed to compare workers in different modalities concerning (a) their sociodemographic characteristics, (b) their working conditions, and (c) workers’ perception of their productivity during the COVID-19 pandemic in Ecuador.

## 2. Materials and Methods

### 2.1. Methods

This study used a quantitative cross-sectional non-experimental exploratory design. A snowball sampling strategy was implemented, mainly with the use of social networks, to collect responses from 633 Ecuadorian adults (over 18 years old) between August 2021 and January 2022. During this time, lockdown measures were more flexible (e.g., no curfew and free mobility within the country), and around 80% of the Ecuadorian population was vaccinated against COVID-19 [48]. However, the Ley Orgánica de Apoyo Humanitario (Organic Law of Humanitarian Support) still applied to some sectors, such as education, which were still online or hybrid, and epidemiological surveillance was still in place. Participants were active in the labor market at any time during the COVID-19 pandemic, either as self-employed or as salaried workers in any work modality. Given the high percentage of informal employment rate in Ecuador, self-employed people were included as a separate category. Participation in this study was voluntary. From the total sample, 91 entries were excluded as participants declared not living in Ecuador at the time of the study, were not working during the pandemic, were working on unremunerated domestic tasks, or were already retired. The final sample constituted 542 participants. The study was conducted following the Declaration of Helsinki and approved by the Ethics Committee of Pontificia Universidad Católica del Ecuador (CEI-09-2021 of 13 August 2021).

### 2.2. Measures

Three categories were assessed using a close-ended question survey:

1. Sociodemographic characteristics: gender, age, area of current habitation, level of education, current relationship status, whether they have children and how many, the number of persons living in their same household, and their current occupation (items 1 through 9);
2. Working conditions: if they had more than one job and the type of employment status in the job in which they spend most of their time, the type of organization they work for, the size and sector of the economy of said organization, the level of business activity, how long they have been in that position, their monthly gross salary if they had a salary reduction and if they developed a new venture (items 10 through 19);
3. Workers’ perceptions about their performance within their company: a feeling of personal and work-related goal accomplishment, changes in working hours, preferences in modalities, the level of satisfaction with the company they currently work for, the state of communication and trust with work colleagues, the perspective on their time management skills, the frequency of work-related objective completion, and the perceived productivity, which refers to a subjective productivity measure of worker outcomes and goals (items 20 through 28). The responses tabulation was developed using Excel spreadsheets; the dataset is available for replication in the Mendeley repository [49].

### 2.3. Data Analysis

The dataset was analyzed using the SPSS statistical software version 25 (IBM, Armonk, NY, USA). In the final database, a missing value on item 20 was replaced with the mode of responses to that item (Mo = 2). Variables were described and then compared by working modalities. The mean, standard deviation, median, and interquartile range were calculated to describe continuous variables. For categorical variables, frequencies and percentages were computed. In order to compare the variables by modality, the Chi-square test was used for categorical variables, the ANOVA test for age, and the Kruskal–Wallis test for the number of people living in the same household. Significance was restricted at *p* < 0.01 to avoid Type 1 error.

## 3. Results

In total, 542 participants completed the survey, of which 57.2% were women. The age range was 18 to 68 years (M = 35.72, SD = 10.35). Most participants were living in an urban area (92.8%), and the majority had at least undergraduate studies (86.2%). Table 1 shows a detailed description of the sociodemographic information of the studied sample by working modality.

After analyzing the differences between modalities, only the variable, education level, presented a significant difference. A higher count of participants (*n* = 48) than expected (*n* = 35) that have elementary or secondary education was evidenced in the on-site modality.

The results showed significant differences in the working conditions according to the monthly gross salary between working modalities. Less (*n* = 49) than the expected count (*n* = 72) of persons who worked on-site modality had a monthly salary greater than 1600 USD; in contrast, more participants (*n* = 68) than the expected (*n* = 46) working in hybrid modality had a monthly salary greater than 1600 USD. Moreover, fewer (*n* = 24) than the expected number of persons (*n* = 31) in teleworking modality had a monthly salary ranging between 401 USD and 800 USD. Of note, some conditions showed a clear tendency, although there were no statistically significant differences. Regarding having more than one job, more (*n* = 105) than the expected (*n* = 41) number of persons were working in the hybrid modality; in the type of organization, more (*n* = 64) than the expected (*n* = 51) were working in the on-site modality in public organizations. Table 2 shows a detailed description of the variables and the statistics testing the differences between modalities for each.

Regarding workers’ perception of their performance and situation at the workplace, several features depend significantly on the working modality. One was the perceived change in the number of hours per workday. In the on-site modality, fewer people (*n* = 105) than the expected (*n* = 144) perceived their working hours had increased; in contrast, more (*n* = 120) than the expected number of workers (*n* = 93) in the hybrid modality perceived that the working hours had increased. Moreover, the preferred type of modality depended on the working modality. More than the expected count of workers in the telework (*n* = 38) and hybrid (*n* = 35) modalities (*n* = 19 and *n* = 26, respectively) prefer only to work in a remote modality, and a higher number of participants (*n* = 145) than the expected count in the on-site modality (*n* = 90) prefer to work in the same on-site modality.

The analysis showed a significant dependency regarding management time ability. There was a higher count of workers (*n* = 19) than expected (*n* = 10) in the hybrid modality who felt they were not able to manage their time. In contrast, more (*n* = 235) than the expected count of workers (*n* = 218) on the on-site modality perceived that they could manage their time. The results also show significant differences in perceived productivity. In the on-site modality, less (*n* = 1) than the expected number of workers did not feel productive (*n* = 4), while less (*n* = 76) than the expected number of workers in the hybrid modality felt productive (*n* = 90). Of note, more than half of the participants were satisfied with the actions taken by their company to face the pandemic (58.7%). Moreover, 49.3% of the workers did not perceive communication problems with others in the company, and the majority (89.3%) of the participants felt that their co-workers trusted them. Table 3 shows detailed information about workers’ perceptions by modality. 

When describing the situation of those who spent at least some of their time working remotely (remote and hybrid modalities), just 20.7% (*n* = 59) indicated that they worked remotely before the pandemic and, among those, almost half of them (49.2%, *n* = 29) had worked frequently in that modality. Most of those performing some remote work (82.5%, *n* = 235) also declared that they had adequate conditions, a specific place (78.6%, *n* = 224), and flexible hours to work (69.8%, *n* = 199). Almost 38% (*n* = 108) worked in a city other than the company’s location, and more than half (56.8%, *n* = 162) declared adapting with ease to remote work.

## 4. Discussion

The COVID-19 pandemic and its consequences changed how people worked momentarily or permanently. Most studies about working conditions focus on a specific modality, while this is one of the few studies comparing the characteristics of the people working in three different modalities: on-site, telework, and hybrid. This exploratory study aimed at filling this gap. Our results contribute to expanding the understanding of the differences between work modalities according to workers’ sociodemographic characteristics, working conditions, and self-perception of productivity.

Concerning sociodemographic characteristics, our results show differences between modalities according to educational levels. A higher proportion of people without higher education worked on-site jobs compared to the other modalities. This result might reflect that occupations with a remote option that requires ICTs are more accessible and require people with higher education [50]. However, the support that our results provide to this conclusion should be taken cautiously since most of our sample (86.2%) had higher education. The sample composition may come as the result of the methods used in this study, which implemented an online survey due to the lack of in-person access to participants, restricting the scope of our findings to those with some access to ICTs. Other sampling methods might help capture information from persons with less access to technologies. Even though we did not find significant differences according to gender, this variable should be considered in future studies since the evidence shows mixed results. For example, a qualitative study with Chilean academics shows that, despite the multiple advantages of remote and hybrid work, there was an increase in workload and shared responsibilities between house duties and regular work [40]. Moreover, an investigation by Utzet and colleagues [13] with 15,070 participants in Spain reflected that female frontline workers face worse work conditions, a high emotional demand, and worse health than women who are not on the frontline. On the other hand, the same study showed that male frontline workers reported lower levels of work-related stress, work–family conflict, and job insecurity.

Regarding our second objective, which explored working conditions, we found a statistically significant difference in monthly gross salary by working modalities. Our results show that people in the hybrid modality earn a higher monthly salary than those in telework and on-site. Similar findings from a study conducted in Germany encountered a positive relationship between a higher mean income and jobs with the opportunity to work remotely [51]. This could also be related to the relationship between jobs with a remote modality option and the need for people with higher education and access to ICTs. However, there is still limited literature on the effect between a specific working modality and the salary a person receives based on each job performed.

There were no other significant differences between work modalities according to working conditions. However, our results reflect a tendency between having more than one job and working in a hybrid modality. This finding is consistent with other studies that show that hybrid workers are more likely to engage in various forms of work or have multiple jobs by taking advantage of digital tools and mobility [52]. Another tendency was evident according to the type of organization. From our findings, it would seem that the proportion of people in the on-site modality is higher in the public sector than in the other modalities. However, this result should be interpreted with caution as there was no statistically significant difference, and the data do not differentiate between the type of activities carried out in each job. Several studies showed that in the public sector, employees in specific areas such as administrative tasks, employees with higher education, and employees earning higher wages are more likely to work from home [53]. In contrast, employees required to work on-site are often in charge of customer interface experience [53]. Future research needs to be conducted on how the type of employment, sector, and the number of jobs a person engages in, relate to each working modality. Furthermore, there is a need to investigate the impact of effective practices implemented by institutions in different economic sectors to increase employee satisfaction in each modality.

Similar to results from another research [54], almost half of our sample reported working remotely. Moreover, most participants who worked remotely reported fair working conditions, having a specific working space, and an easy adaptation to this working modality. These results are similar to recent studies that found positive consequences in countries that transitioned to this modality in response to the COVID-19 pandemic [35]. Another study in Kuwait showed that most teleworkers appreciated the flexibility in setting work schedules and reported satisfaction with the company’s immediate response to COVID-19 remote working conditions [36]. Comparably, a study from South Africa shows that teleworkers have a better work–life balance and save more money [37]. Income and lifestyle might be related to the ability to adapt to telework [42]. Future studies should investigate actions taken by the companies to facilitate the transition to remote working during the pandemic and analyze which responses have better outcomes.

By contributing to the existing literature, our last objective was to examine workers’ perceptions of their productivity during this time in Ecuador. Our results suggest that perceived productivity varied according to work modality. Contrary to participants in the on-site modality, people in the hybrid modality perceived a decrease in productivity, an increase in daily working hours, and fewer time-management abilities. Businesses and institutions need to understand the relationship between productivity and work modality because previous research on satisfaction and productivity during the pandemic has had mixed results [18,21,24,36,37]. Regarding other working conditions, such as the sense of accomplishment of goals and objectives, satisfaction with their institutions’ actions to face the pandemic, quality of communication with peers, and colleagues’ trust, the current study did not find any significant differences in modalities. Further research must deepen the relationship between work modality and productivity with other qualitative and quantitative measures for productivity.

This study is not without limitations. Data collection through an online survey limits participation to those with internet access and an electronic device to complete the survey. Therefore, we poorly captured the experience of people living in rural areas or with no access to the internet. Although the final sample is adequate, future studies should include more participants in each modality. Finally, most of our participants worked in the tertiary business sector. Providing larger samples and promoting the inclusion of participants in the primary and secondary sectors might provide more information about their reality, working conditions, and workers’ perceptions, as well as comparisons between sectors, further helping to understand their particularities. Nevertheless, these findings support other studies and show that the opportunity to transition to other modalities depends on the nature of each specific task [14].

## 5. Conclusions

The present study identified and compared working conditions and workers’ perceptions of productivity among on-site workers, teleworkers, and hybrid workers in Ecuador during the COVID-19 pandemic. This is one of the few studies comparing working modalities that provide valuable information for decision-makers in organizations considering switching or maintaining one or several of those modalities. The results show that a higher proportion of people without higher education worked on-site jobs compared to the other modalities. Regarding monthly income, people in the hybrid modality earned a higher salary than those in telework and on-site. Almost half of our sample reported working remotely with fair working conditions, specific working space, and good adaptation to this modality. Interestingly, people in the hybrid modality perceived a decrease in productivity, an increase in daily working hours, and less time-management abilities. However, most people in the hybrid modality would like to stay in that modality, and almost half of teleworkers would prefer to switch to it. Based on our findings, we suggest that institutions: (1) take into count workers’ productivity and preferences when making decisions on the implementation of new work modalities; (2) look for different measures to mitigate the impact of the pandemic besides salary reductions; (3) design strategies for better time management, especially for workers in telework and hybrid modality; and (4) collaborate with academia to gain a better understanding of the working conditions that were effective during the pandemic in different economic sectors for creating future contingency plans.

## Figures and Tables

**Table 1:** Sociodemographic characteristics by working modality and comparison statics.

Sociodemographic Characteristics	On-Site	Telework	Hybrid	Total	Statistics		
	(*n* = 257)	(*n* = 119)	(*n* = 166)	(*n* = 542)	*X*^2^/*F*/*H*	*df*	*p*
Gender					4.29	4	0.37
Men	115 (44.7%)	44 (37%)	70 (42.2%)	229 (42.3%)			
Women	142 (55.3%)	75 (63%)	95 (57.2%)	312 (57.6%)			
Non-binary	0 (0%)	0 (0%)	1 (0.6%)	1 (0.2%)			
Age	34.98 (SD = 9.83)	34.97 (SD = 10.72)	37.39 (SD = 10.69)	35.72 (SD = 10.35)	3.15	2	0.04
Area					0.96	2	0.62
Urban	240 (93.4%)	108 (90.8%)	155 (93.4%)	503 (92.8%)			
Rural	17 (6.6%)	11 (9.2%)	11 (6.6%)	39 (7.2%)			
Education					10.5	2	0.005 *
Not higher education	48 (18.7%)	14 (11.8%)	13 (7.8%)	75 (13.8%)			
Higher education	209 (81.3%)	105 (88.2%)	153 (92.2%)	467 (86.2%)			
Relationship status					5.25	4	0.26
No stable relationship	75 (29.2%)	43 (36.1%)	45 (27.1%)	163 (30.1%)			
Stable relationship, not living together	68 (26.5%)	26 (21.8%)	35 (21.1%)	129 (23.8%)			
Stable relationship, living together	114 (44.4%)	50 (42%)	86 (51.8%)	250 (46.1%)			
Children					0.54	2	0.97
No	136 (52.9%)	64 (53.8%)	87 (52.4%)	287 (53%)			
Yes	121 (47.1%)	55 (46.2%)	79 (47.6%)	255 (47%)			
Number of hours helping children with school tasks per day					1.9	4	0.75
(*n* = 255)							
Not supporting	52 (20.2%)	23 (19.3%)	28 (16.9%)	103 (19%)			
Up to 3 h	54 (21.0%)	26 (21.8%)	43 (25.9%)	123 (22.7%)			
More than 3 h	15 (5.8%)	6 (5%)	8 (4.8%)	29 (5.4%)			
Number of people living in the household	Me = 3 (2–4)	Me = 3 (2–4)	Me = 2.5 (1–3)	Me = 3 (1–4)	2.81	2	0.61
Occupation					6.92	2	0.31
Working and studying	74 (28.8%)	43 (36.1%)	68 (41%)	185 (34.1 %)			
Working	183 (71.2%)	76 (63.9%)	98 (59%)	357 (65.9%)			

Note. * *p* < 0.01.

**Table 2:** Working conditions during the pandemic by working modality and comparison statistics.

Working Conditions	On-Site	Telework	Hybrid	Total	Statistics		
	(*n* = 257)	(*n* = 119)	(*n* = 166)	(*n* = 542)	*X*^2^/*F*/*H*	*df*	*p*
More than one job					10.29	2	0.01
No	197 (76.7%)	91 (76.5%)	105 (63.3%)	393 (72.5%)			
Yes	60 (23.3%)	28 (23.5%)	61 (36.7%)	149 (27.5%)			
Type of employment status (a job for which you spend most of the time)					8.17	4	0.09
Employment	212 (82.5%)	39 (15.2%)	118 (71.1%)	424 (78.2%)			
Self-employment	39 (15.2%)	22 (18.5%)	40 (24.1%)	101 (18.6%)			
Informal job	39 (15.2%)	3 (2.5%)	8 (4.8%)	17 (3.1%)			
Type of organization					8.58	2	0.01
Private and NGO	193 (75.1%)	96 (80.7%)	144 (86.7%)	433 (79.9%)			
Public	64 (24.9%)	23 (19.3%)	22 (13.3%)	109 (20.1%)			
Size of the organization					1.19	6	0.1
Micro (1 to 9 individuals)	42 (16.3 %)	17 (14.3%)	31 (18.7%)	90 (16.6%)			
Small (10 to 49 individuals)	36 (14%)	16 (13.4%)	22 (13.3%)	74 (13.7%)			
Medium (50 to 199 individuals)	30 (11.7%)	16 (13.4%)	20 (12%)	66 (12.2%)			
Large (over 200 individuals)	149 (58%)	70 (58.8%)	93 (56%)	312 (57.6%)			
Business enterprise sector					10.17	4	0.38
Primary sector	16 (6.2%)	7 (5.9%)	8 (4.8%)	31 (5.7%)			
Secondary sector	38 (14.8%)	8 (6.7%)	11 (6.6%)	57 (10.5%)			
Tertiary sector	203 (79%)	104 (87.4%)	147 (88.6%)	454 (83.8%)			
Levels of business activities					2.77	2	0.25
Operational/Administrative	182 (70.8%)	85 (71.4%)	106 (63.9%)	373 (68.8%)			
Tactical/Strategic	75 (29.2%)	34 (28.6%)	60 (36.1%)	169 (31.2%)			
Time working in the company					11.38	6	0.08
Less than one year	64 (24.9%)	33 (27.7%)	29 (17.5%)	126 (23.2%)			
1 to 3 years	76 (29.6%)	32 (26.9%)	60 (36.1%)	168 (31%)			
4 to 6 years	58 (22.6%)	18 (15.1%)	28 (16.9%)	104 (19.2%)			
More than 7 years	59 (23%)	36 (30.3%)	49 (29.5%)	144 (26.6%)			
Monthly gross salary (USA Dollars)					31.59	6	0.001 *
Less than 400 USD	31 (12.1%)	18 (15.1%)	23 (13.9%)	72 (13.3%)			
Between 401 USD and 800 USD	87 (33.9%)	24 (20.2%)	31 (18.7%)	142 (26.2%)			
Between 801 USD and 1600 USD	90 (35%)	42 (35.3%)	31 (18.7%)	176 (32.5%)			
More than 1600 USD	49 (19.1%)	35 (29.4%)	68 (41%)	152 (28%)			
Salary reduction due to the pandemic					3.04	2	0.22
No	146 (56.8%)	76 (63.9%)	89 (53.6%)	311 (57.4%)			
Yes	111 (43.2%)	43 (36.1%)	77 (46.4%)	231 (42.6%)			
New ventures (e.g., food preparation, offering services, etc.)					0.97	2	0.61
No	173 (67.3%)	81 (68.1%)	105 (63.3%)	359 (66.2%)			
Yes	84 (32.7%)	38 (31.9%)	61 (36.7%)	183 (33.8%)			

Notes. * *p* < 0.01 Economic sector (1) Primary sector refers to the extraction of raw materials. (2) Secondary sector refers to the production or manufacture of finished products. (3) Tertiary sector refers to the supply of intangible goods and services to consumers.

**Table 3:** Workers’ perceptions about their performance within their company by modality and comparison statistics.

Workers’ Perception	On-Site	Telework	Hybrid	Total	Statistics		
	(*n* = 257)	(*n* = 119)	(*n* = 166)	(*n* = 542)	*X* ^2^	*df*	*p*
I feel I have accomplished all my goals					3.41	2	0.18
No	20 (7.8%)	13 (10.9%)	22 (13.3%)	55 (10.1%)			
Yes	237 (92.2%)	106 (89.1%)	144 (86.7%)	487 (89.9%)			
I consider that the number of working hours per day					52.19	4	<0.001 *
have increased	105 (40.9%)	80 (67.2%)	120 (72.3%)	305 (56.3%)			
have not changed	124 (48.2%)	27 (22.7%)	32 (19.3%)	183 (33.8%)			
have been reduced	28 (10.9%)	12 (10.1%)	14 (8.4%)	54 (10%)			
If it was your choice, which of the following options reflects your desired working modality?					116.3	4	<0.001 *
Only remote	14 (5.4%)	38 (31.9%)	35 (21.1%)	87 (16.5%)			
Hybrid	98 (38.1%)	62 (52.1%)	105 (63.3%)	265 (48.9%)			
Only in-person	145 (56.4%)	19 (16%)	26 (15.7%)	190 (35.1%)			
Considering the actions taken by the place where you work, how satisfied do you feel?					15.28	8	0.05
Very unsatisfied	23 (14%)	11 (9.2%)	15 (9%)	62 (11.4%)			
Unsatisfied	43 (16.7%)	20 (16.8%)	18 (10.8%)	81 (14.9%)			
Indifferent	33 (12.8%)	19 (16%)	29 (17.5%)	81 (14.9%)			
Satisfied	93 (36.2%)	42 (35.3%)	81 (48.8%)	216 (39.9%)			
Very satisfied	52 (20.2%)	27 (22.7%)	23 (13.9%)	102 (18.8%)			
How frequently have you had communication and/or organizational problems with the people you work with?					16.15	8	0.04
Never	41 (16%)	12 (4.7%)	12 (7.2%)	65 (12%)			
Rarely	96 (37.4%)	45 (37.8%)	61 (36.7%)	202 (37.3%)			
Sometimes	80 (31.1%)	36 (30.3%)	67 (40.4%)	183 (33.8%)			
Frequently	28 (10.9%)	22 (18.5%)	16 (9.6%)	66 (12.2%)			
Very frequently	12 (4.7%)	4 (3.4%)	10 (6%)	26 (4.8%)			
I believe that the people I work with trust me					17.25	8	0.03
Totally disagree	1 (0.4%)	0 (0%)	3 (1.8%)	4 (0.7%)			
Disagree	2 (0.8%)	0 (0%)	6 (3.6%)	8 (1.5%)			
Indifferent	16 (6.2%)	12 (10.1%)	18 (10.8%)	46 (8.5%)			
Agree	100 (38.9%)	37 (31.1%)	56 (33.7%)	193 (35.6%)			
Totally agree	138 (53.7%)	70 (58.8%)	83 (50%)	291 (53.7%)			
I can manage my time when I am working					22.07	8	0.005 *
Totally disagree	1 (0.4%)	1 (0.8%)	2 (1.2%)	4 (0.7%)			
Disagree	6 (2.3%)	3 (2.5%)	17 (10.2%)	29 (5.4%)			
Indifferent	15 (5.8%)	13 (10.9%)	21 (12.7%)	49 (9%)			
Agree	112 (43.6%)	43 (36.1%)	55 (33.1%)	211 (38.9%)			
Totally agree	123 (47.9%)	59 (49.6%)	71 (42.8%)	249 (45.9%)			
I consider that I am productive in my work					37.6	8	<0.001 *
Totally disagree	1 (0.4%)	1 (0.8%)	0 (0%)	2 (0.4%)			
Disagree	0 (0%)	3 (2.5%)	9 (5.4%)	12 (2.2%)			
Indifferent	6 (2.3%)	13 (10.9%)	20 (12%)	39 (7.2%)			
Agree	89 (34.6%)	43 (36.1%)	61 (36.7%)	193 (54.6%)			
Totally agree	161 (62.6%)	59 (49.6%)	76 (45.8%)	296 (54.6%)			
How often do I achieve my working objectives?					7.64	6	0.27
Never	0 (0%)	0 (0%)	0 (0%)	0 (0%)			
Rarely	1 (0.4%)	1 (0.8%)	1 (0.6%)	3 (0.6%)			
Sometimes	2 (0.8%)	4 (3.4%)	8 (4.8%)	14 (2.6%)			
Frequently	118 (45.9%)	49 (41.2%)	71 (42.8%)	238 (43.9%)			
Very frequently	136 (52.9%)	65 (54.6%)	86 (51.8%)	287 (53%)			

Note. * *p* < 0.01.

## Data Availability

The dataset is accessible through the Mendeley repository (https://doi:10.17632/bp4vdd53w3.1, accessed on 15 August 2022). All other information is included in the article.
